# Prolonged TNF-α stimulation induces a PD-1–associated exhaustion-like phenotype in mesenchymal stromal cells

**DOI:** 10.3389/fcell.2026.1680076

**Published:** 2026-03-06

**Authors:** Naoya Matsunaga, Kentaro Akiyama, Aung Ye Mun, Tingling Zou, Kazuki Ito, Ruji Tagashira, Takuo Kuboki

**Affiliations:** 1 Department of Oral Rehabilitation and Regenerative Medicine, Okayama University Graduate School of Medicine, Dentistry and Pharmaceutical Sciences, Okayama, Japan; 2 Department of Occlusal and Oral Functional Rehabilitation, Okayama University Graduate School of Medicine, Dentistry and Pharmaceutical Sciences, Okayama, Japan

**Keywords:** immune checkpoint, immunoregulatory dysfunction, mesenchymal stromal cells (MSCs), prolonged inflammatory stimulation, TNF-α (tumor necrosis factor-alpha)

## Abstract

Mesenchymal stromal cells (MSCs) have emerged as promising therapeutic agents for inflammatory diseases because of their potent immunomodulatory properties. Although acute inflammation transiently enhances MSC functionality, the impact of chronic inflammatory exposure remains poorly defined. In this study, we investigated the effects of sustained TNF-α stimulation and indirect co-culture with M1 macrophages on MSC behavior. Comprehensive gene expression profiling was performed to assess the changes in immunoregulatory, apoptotic, and metabolic pathways. To determine functional reversibility, we also evaluated MSCs following the withdrawal of TNF-α. Short-term exposure led to upregulation of *Tgf-β*, *Il-10*, and *Fasl*, whereas prolonged stimulation suppressed these genes and significantly increased the expression of immune checkpoint genes *Pd-1* and *Ctla-4*, indicative of an exhaustion-like phenotype. This phenotypic shift was associated with sustained NF-κB activation, upregulation of *Stat3* and *Ap-1*, suppression of mTORC1/2 components, decreased *Pd-l1* expression, and increased *Pd-1* expression, raising the possibility that PD-1 upregulation is associated with MSC dysfunction under chronic inflammatory stress. These findings revealed that prolonged stimulation (48 h) induces an exhaustion-like dysfunction state in MSCs, characterized by checkpoint activation, transcriptional repression, and metabolic dysfunction. PD-1 may serve as a biomarker associated with inflammation-induced MSC impairment.

## 1 Introduction

Mesenchymal stromal cells (MSCs) are multipotent cells present in various tissues (Horwitz et al., 2005; da Silva et al., 2006) and are widely recognized for their immunomodulatory properties (Caires et al., 2017; Petrini et al., 2009; González et al., 2009; Akiyama et al., 2012; Shen et al., 2021). Although transient inflammatory stimuli enhance MSC function, persistent inflammatory activation, as observed in autoimmune disorders, may compromise their regulatory capacity. This study investigated whether prolonged exposure to tumor necrosis factor-alpha (TNF-α) impairs MSC function, resembling an exhaustion-like state characterized by sustained expression of immune checkpoint molecules and reduced immunoregulatory gene expression.

Our previous research demonstrated that MSCs co-cultured with M1-polarized macrophages upregulate immunomodulatory genes, and that macrophage depletion impairs MSC accumulation at wound sites and delays tissue repair. These effects appear to be mediated, at least in part, by TNF-α derived from M1 macrophages, which promote MSC-driven tissue regeneration (Mun et al., 2024). These findings support the concept of an inflammation-regeneration axis, wherein transient inflammatory signaling facilitates MSC-mediated repair. In contrast, chronic antigenic stimulation, as seen in persistent viral infections and malignancies, leads to T cell exhaustion, characterized by upregulation of PD-1 and CTLA-4, disruption of downstream signaling, and diminished effector function (Wherry et al., 2007; Zheng et al., 2022). Similar mechanisms have been described in tumor-infiltrating lymphocytes, where PD-L1 expression on cancer cells further impairs T cell metabolism and cytotoxicity (Gunasinghe et al., 2021).

Similarly, chronic inflammatory or antigenic stimulation may compromise the immunomodulatory function of MSCs. For instance, prolonged cytokine exposure during chronic inflammation or systemic sclerosis diminishes the osteogenic potential of MSCs and contributes to osteoporotic phenotypes (Chen et al., 2015). However, the effects of sustained inflammation on the immunomodulatory functions of MSCs remain poorly understood.

The present study aims to elucidate how sustained inflammatory signals affect MSC function, with a focus on the concept of “immunomodulatory exhaustion-like phenotype.” Although PD-1 is a well-established immune checkpoint receptor in T cells, its inducible expression and functional relevance in MSCs remain unclear. We hypothesized that sustained inflammatory stimulation could induce PD-1 expression in MSCs, concomitant with a decline in immunoregulatory gene expression. A deeper understanding of this mechanism may provide a conceptual framework for future studies investigating tissue damage and regeneration under chronic inflammatory conditions.

## 2 Materials and methods

### 2.1 Animals

All animal procedures were approved by the Animal Experiment Committee of Okayama University (approval numbers OKU-2018189 and 2021377). Female C57BL/6J mice (10 weeks old; CLEA Japan, Tokyo, Japan) were euthanized via cervical dislocation, and the femurs were harvested. To induce sustained inflammatory conditions, lipopolysaccharide (LPS) derived from *Porphyromonas gingivalis* (LPS-PG; 5 mg/kg; InvivoGen, CA, United States) was administered intraperitoneally. Femurs were collected on day 7, and real-time PCR was used to assess the expression of *Tnf-α* and *iNos* in the bone marrow as indicators of inflammation.

### 2.2 MSCs and macrophages culture

Bone marrow cells were isolated from the femurs and tibiae and filtered through a 70 μm cell strainer (Greiner Bio-One, Kremsmünster, Austria). The cells were washed with PBS containing 2% fetal bovine serum (FBS) and cultured in α-MEM supplemented with 15% FBS, 2 mM L-glutamine, 100 U/mL penicillin-streptomycin, and 55 µM 2-mercaptoethanol (Life Technologies, CA, United States) at 37 °C in 5% CO_2_. After 14 days, colony-forming units were passaged, and cells at passage 2 were used in the experiments. The characterization of MSCs at passage 2 is provided in the Supplementary Material.

Bone marrow-derived macrophages were cultured in DMEM containing 10% FBS, 100 U/mL penicillin-streptomycin, and 100 ng/mL M-CSF (BioLegend, CA, United States) for 5 days. M1 polarization was induced by treating cells with 20 ng/mL LPS and 50 ng/mL IFN-γ for 12 h.

**FIGURE 1:**
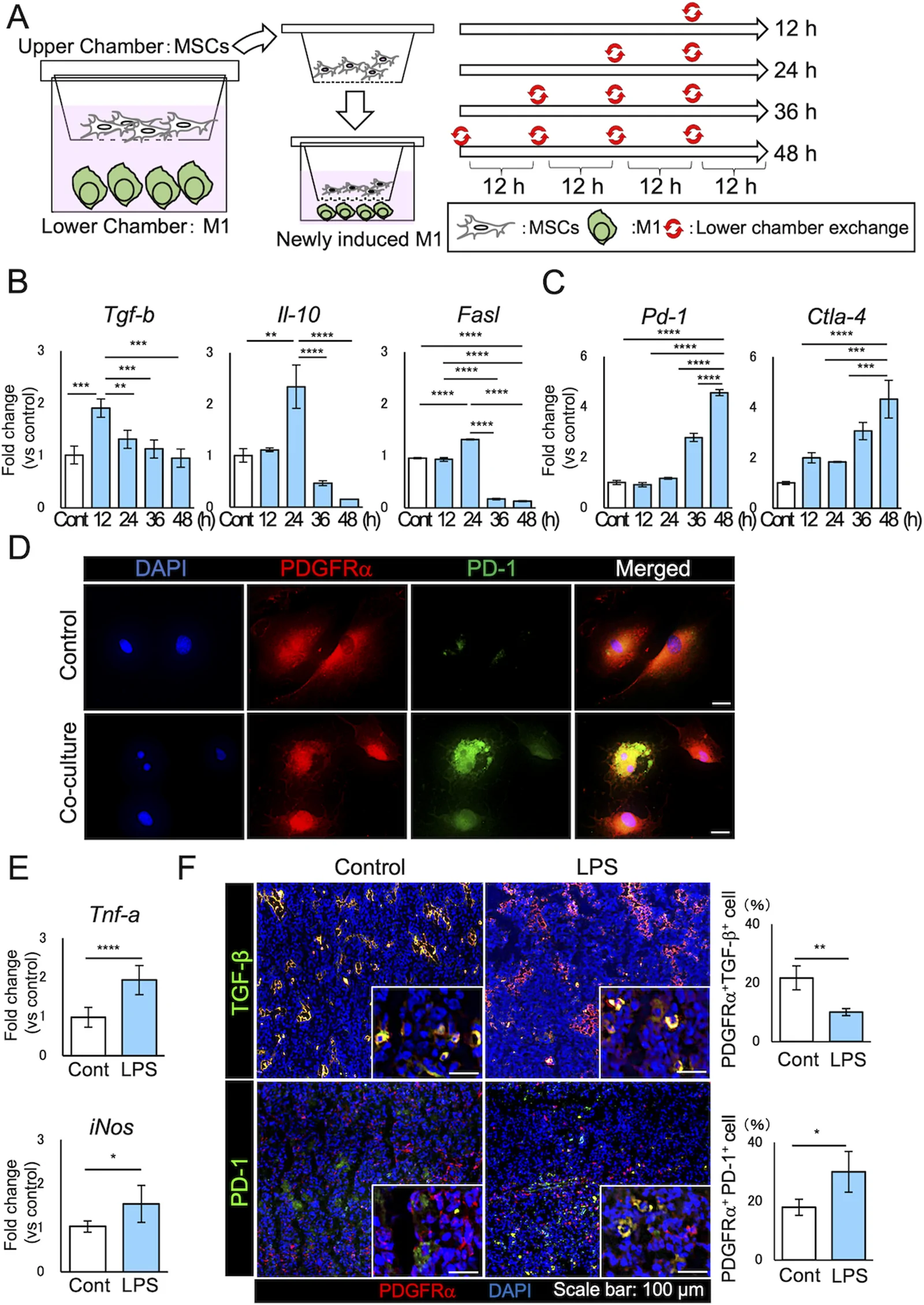
Immunomodulatory exhaustion-like phenotype of MSCs under prolonged TNF-α stimulation. **(A)** Schematic representation of indirect co-culture system. MSCs were repeatedly exposed to inflammatory stimulation by transferring them to freshly polarized M1 macrophages every 12 h using Transwell inserts. **(B)** Time-course analysis of immunoregulatory gene expression (*Tgf-β*, *Il-10*, and *Fasl*) in MSCs during co-culture. *Tgf-β* expression peaked at 12 h, while *Il-10* and *Fasl* were transiently upregulated and then declined. **(C)** Progressive increase in the expression of immune checkpoint genes (*Pd-1* and *Ctla-4*) over time. **(D)** Representative immunofluorescence images showing co-expression of PDGFRα and PD-1 in MSCs after 48 h of co-culture with M1 macrophages (Scale bar = 20 µm). Images are representative of independent experiments and are shown to illustrate the spatial distribution and co-expression of PDGFRα and PD-1, rather than serving as quantitative evidence. **(E)** Increased *Tnf-α* and *iNos* expression in the bone marrow following intraperitoneal injection of LPS-PG (5 mg/kg) for 7 days. **(F)** Immunohistochemistry of bone marrow showing decreased PDGFRα^+^TGF-β^+^ cells and increased PDGFRα^+^PD-1^+^ cells in LPS-treated mice compared to untreated controls (Scale bar = 100 µm). Quantitative analysis was performed at this magnification, which allowed for the reliable identification of PD-1–positive MSC-like cells. Data represent mean ± SD of independent biological replicates derived from separately prepared MSC cultures (n = 4–5). One-way ANOVA with Tukey’s *post-hoc* test was used. *p < 0.05, **p < 0.01, ***p < 0.001, ****p < 0.0001.

For indirect *in vitro* co-culture, MSCs and M1 macrophages were co-cultured (MSCs:M1 = 1:1) in a Transwell system (0.4 µm pore size; Corning, NY, United States), allowing cytokine exchange without direct cell-to-cell contact. MSCs were transferred to freshly induced M1 macrophages every 12 h to ensure continuous inflammatory stimulation (Figure 1A). The co-culture durations were set at 12, 24, 36, and 48 h. Total RNA was extracted from the MSCs at each time point.

### 2.3 TNF-α neutralization

To assess the role of TNF-α secreted by M1 macrophages, a TNF-α neutralizing antibody (Cell Signaling Technology, MA, United States) was added to the MSC culture medium at a final concentration of 1 μg/mL during co-culture experiment. Control cultures were maintained without antibody treatment, which showed no detectable difference compared with isotype control conditions in preliminary experiments. Gene expression at 12 and 48 h was compared with that of the controls cultured without the neutralizing antibody.

### 2.4 TNF-α stimulation

MSCs were stimulated with recombinant TNF-α at a concentration determined by preliminary testing that yielded reproducible responses without excessive cell loss (10 ng/mL; Invitrogen, CA, United States) for 12–48 h, with the medium changed every 12 h. Preliminary dose-ranging experiments were performed to balance inflammatory responsiveness and cell viability, leading us to select 10 ng/mL TNF-α for subsequent experiments. For recovery experiments, TNF-α was withdrawn after 36 h, and MSCs were subsequently cultured in TNF-α–free medium. Samples were collected at defined intervals after withdrawal (Figure 4A).

### 2.5 Immunocytochemistry (ICC)

MSCs were fixed with 4% paraformaldehyde (VWR International, PA, United States), blocked with 5% goat serum (Life Technologies, CA, United States), and incubated with primary antibodies against PDGFRα (1:100; Life Technologies), TGF-β1 (1:100; Life Technologies), PD-1 (1:400; Cell Signaling Technology), IL-10 (1:100; Abcam, Cambridge, United Kingdom), and Caspase-3 (1:100; Cell Signaling Technology). Alexa Fluor®–conjugated secondary antibodies (Invitrogen) were used for detection. The nuclei were counterstained with DAPI (Life Technologies). Immunofluorescence quantification was performed using the ImageJ software (National Institutes of Health, Bethesda, MD, United States). Positive cells were defined as those with fluorescence intensity above background levels, and the percentage of positive cells was calculated from multiple independent fields.

### 2.6 Gene expression analysis (real-time PCR)

Total RNA was extracted using the PureLink RNA Mini Kit (Life Technologies). cDNA synthesis was performed using the iScript cDNA Synthesis Kit (Bio-Rad, Hercules, CA, United States), and quantitative PCR was performed using the KAPA SYBR FAST qPCR Master Mix (KAPA BIOSYSTEMS, Wilmington, MA, United States). Primer sequences are listed in the Supplementary Table.

### 2.7 Immunohistochemistry (IHC)

Femurs were fixed in 4% paraformaldehyde, cryo-embedded, and sectioned to a thickness of 5 µm. The sections were stained with primary antibodies against PD-1 (1:400; Cell Signaling Technology) and PDGFRα (1:100; R&D Systems, MN, United States), followed by Alexa Fluor®–conjugated secondary antibodies (Life Technologies). The nuclei were counterstained with DAPI.

### 2.8 Statistical analysis

Statistical analyses were performed using one-way analysis of variance (ANOVA), followed by Tukey’s multiple comparisons test. GraphPad Prism 9 software (GraphPad Software, San Diego, CA, United States) was used for statistical analysis. Data are presented as mean ± standard deviation (SD).

## 3 Results

### 3.1 Prolonged inflammatory stimulation reduces the immunomodulatory capacity of MSCs and induces an exhaustion-like phenotype

At passage 2, MSCs displayed a typical surface marker profile (CD140a^+^, Sca-1^+^, CD146^+^, CD105^+^, CD34^−^, and CD14^−^) and retained osteogenic and adipogenic differentiation capacities, as confirmed by both gene expression and histological staining (Supplementary Figure S1). Heterogeneity at this early passage was minimal, based on the variability in marker expression. Following indirect co-culture with M1 macrophages, MSCs exhibited a time-dependent decline in immunomodulatory gene expression. *Tgf-β* expression peaked at 12 h, whereas *Il-10* and *Fasl* were transiently upregulated between 12 and 24 h, followed by marked downregulation after 36 h (Figure 1B). In contrast, the expression of the immune checkpoint genes *Pd-1* and *Ctla-4* progressively increased over time (Figure 1C). Immunocytochemistry revealed double-positive cells for PDGFRα and PD-1 in MSCs co-cultured with M1 macrophages (Figure 1D). In a murine model of sustained inflammatory conditions induced by LPS-PG injection, bone marrow expression of *Tnf-α* and *iNos* was elevated (Figure 1E). Moreover, the number of PDGFRα^+^TGF-β^+^ cells in the bone marrow was reduced in LPS-treated mice compared to that in the untreated controls, whereas the number of PDGFRα^+^PD-1^+^ cells increased (Figure 1F).

### 3.2 TNF-α neutralization attenuates M1 macrophage–associated exhaustion-like features in MSCs

**FIGURE 2:**
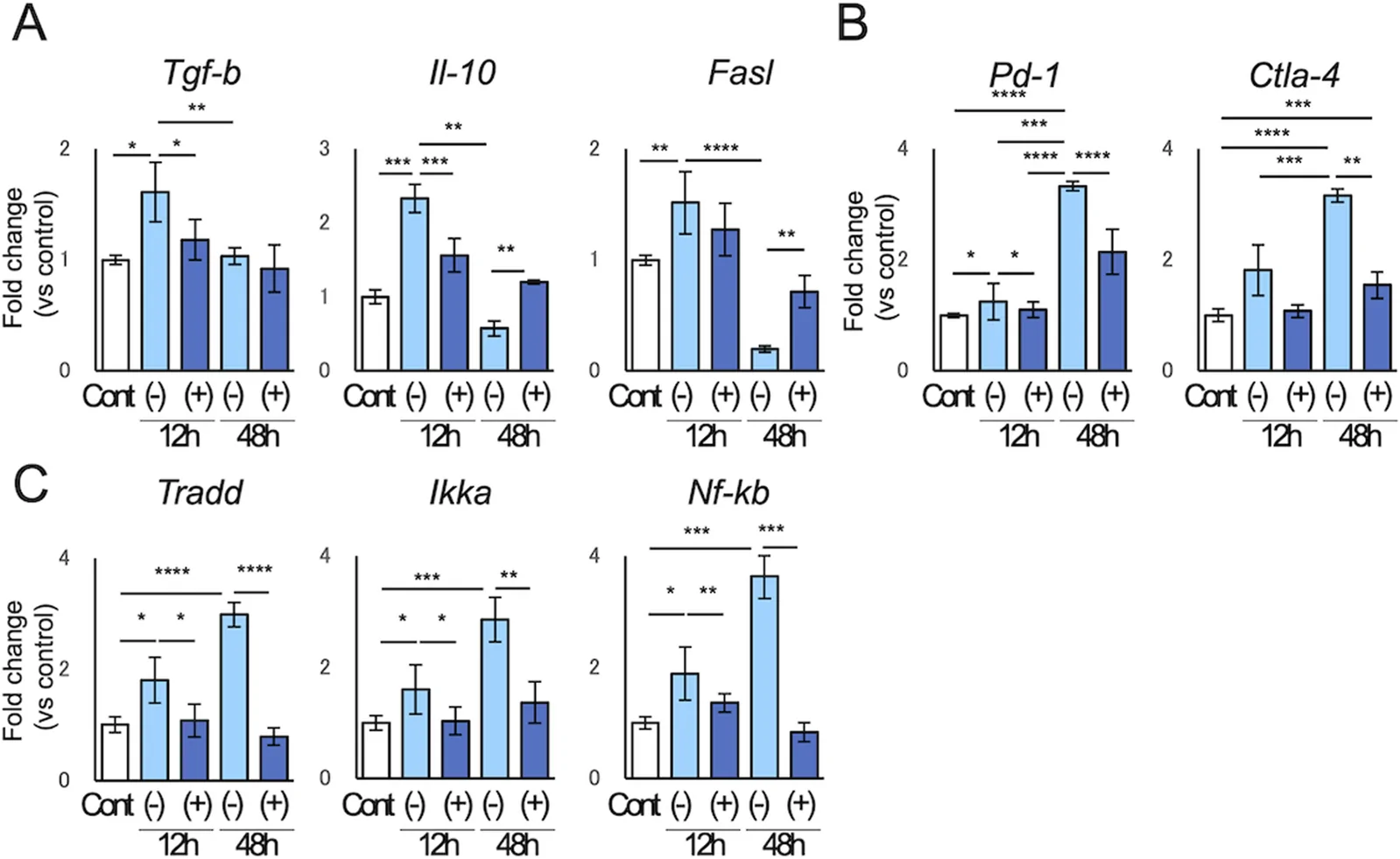
TNF-α neutralization partially restores immunoregulatory gene expression in MSCs. **(A)** Expression of *Tgf-β*, *Il-10*, and *Fasl* in MSCs co-cultured with M1 macrophages for 12 and 48 h, with or without TNF-α neutralizing antibody. Neutralization inhibited early upregulation (12 h) and prevented the subsequent downregulation observed at 48 h. **(B)** TNF-α neutralization suppressed the late-phase induction of *Pd-1* and *Ctla-4*. **(C)** Expression of TNF-α signaling-related genes (*Tradd*, *Ikkα*, and *Nf-κb*) was also reduced after antibody treatment. Data represent mean ± SD of independent biological replicates derived from separately prepared MSC cultures (n = 4–5). One-way ANOVA with Tukey’s *post-hoc* test was used. *p < 0.05, **p < 0.01, ***p < 0.001, ****p < 0.0001.

To determine whether the observed changes in MSCs were mediated by TNF-α derived from M1 macrophages, we introduced a TNF-α–neutralizing antibody during co-culture. This intervention attenuated the early upregulation of *Tgf-β* and *Il-10* at 12 h and prevented the downregulation of *Il-10* and *Fasl* at 48 h (Figure 2A). Furthermore, late-phase induction of *Pd-1* and *Ctla-4* was significantly inhibited by TNF-α neutralization (Figure 2B), along with reduced expression of downstream signaling components *Tradd*, *Ikkα*, and *Nf-κb* (Figure 2C).

### 3.3 Sustained TNF-α stimulation induces MSC exhaustion-like phenotype and suppresses immunoregulatory genes

**FIGURE 3:**
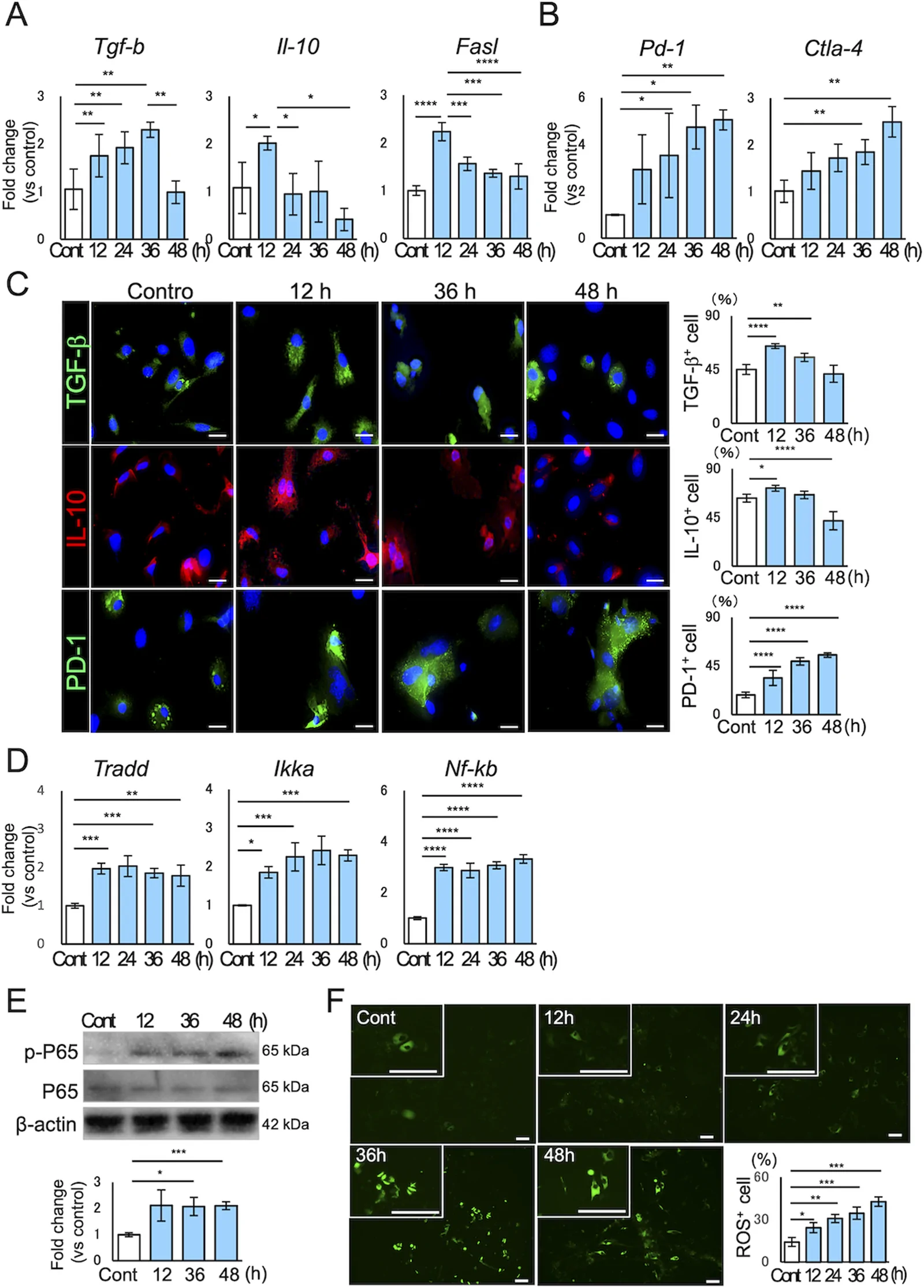
Prolonged TNF-α stimulation induces an exhaustion-like phenotype in MSCs. **(A)** Time-course expression of *Tgf-β*, *Il-10*, and *Fasl* in MSCs stimulated with TNF-α (10 ng/mL) for up to 48 h. An initial upregulation at the indicated time points was observed, followed by significant downregulation. **(B)** Immune checkpoint genes (*Pd-1* and *Ctla-4*) were progressively upregulated over time. **(C)** Immunofluorescence revealed decreased numbers of TGF-β^+^ and IL-10^+^ MSCs and increased PD-1^+^ MSCs with prolonged TNF-α stimulation (Scale bar = 20 µm). **(D)** Expression of *Tradd*, *Ikkα*, and *Nf-κb* remained elevated throughout stimulation. **(E)** Western blot analysis showed sustained phosphorylation of p65 in MSCs treated with TNF-α. Quantification was performed by densitometric analysis of independent biological replicates (n = 3), each derived from separately prepared MSC cultures. **(F)** Prolonged TNF-α stimulation led to an increased number of ROS-positive MSCs, indicating elevated intracellular oxidative stress (Scale bar = 100 µm). ROS positivity was defined using a fixed fluorescence intensity threshold, and all samples were processed in parallel, under identical acquisition settings. ROS data are presented as supportive evidence rather than definitive mechanistic proof. Data represent mean ± SD of independent biological replicates derived from separately prepared MSC cultures (n = 4–5). One-way ANOVA with Tukey’s *post-hoc* test was used. *p < 0.05, **p < 0.01, ***p < 0.001, ****p < 0.0001.

Direct stimulation of MSCs with TNF-α recapitulated the exhaustion-like phenotype observed in the co-culture system. *Tgf-β*, *Il-10*, and *Fasl* expression was transiently upregulated, peaking at 12–24 h and declining thereafter (Figure 3A). Conversely, *Pd-1* and *Ctla-4* expression increased steadily over the entire 48-h stimulation period (Figure 3B). Immunocytochemical analysis confirmed this trend: TGF-β^+^ and IL-10^+^ MSCs peaked early and subsequently declined, whereas PD-1^+^ MSCs increased over time (Figure 3C). Notably, *Tradd*, *Ikkα*, and *Nf-κb* levels remained elevated throughout the stimulation period (Figure 3D), and Western blotting revealed the sustained phosphorylation of p65 (Figure 3E), consistent with sustained NF-κB signaling. In addition, the number of Reactive Oxygen Species (ROS)-positive MSCs increased progressively over the stimulation period, indicating sustained intracellular oxidative stress (Figure 3F).

### 3.4 MSC dysfunction persists even after withdrawal of inflammatory stimuli

**FIGURE 4:**
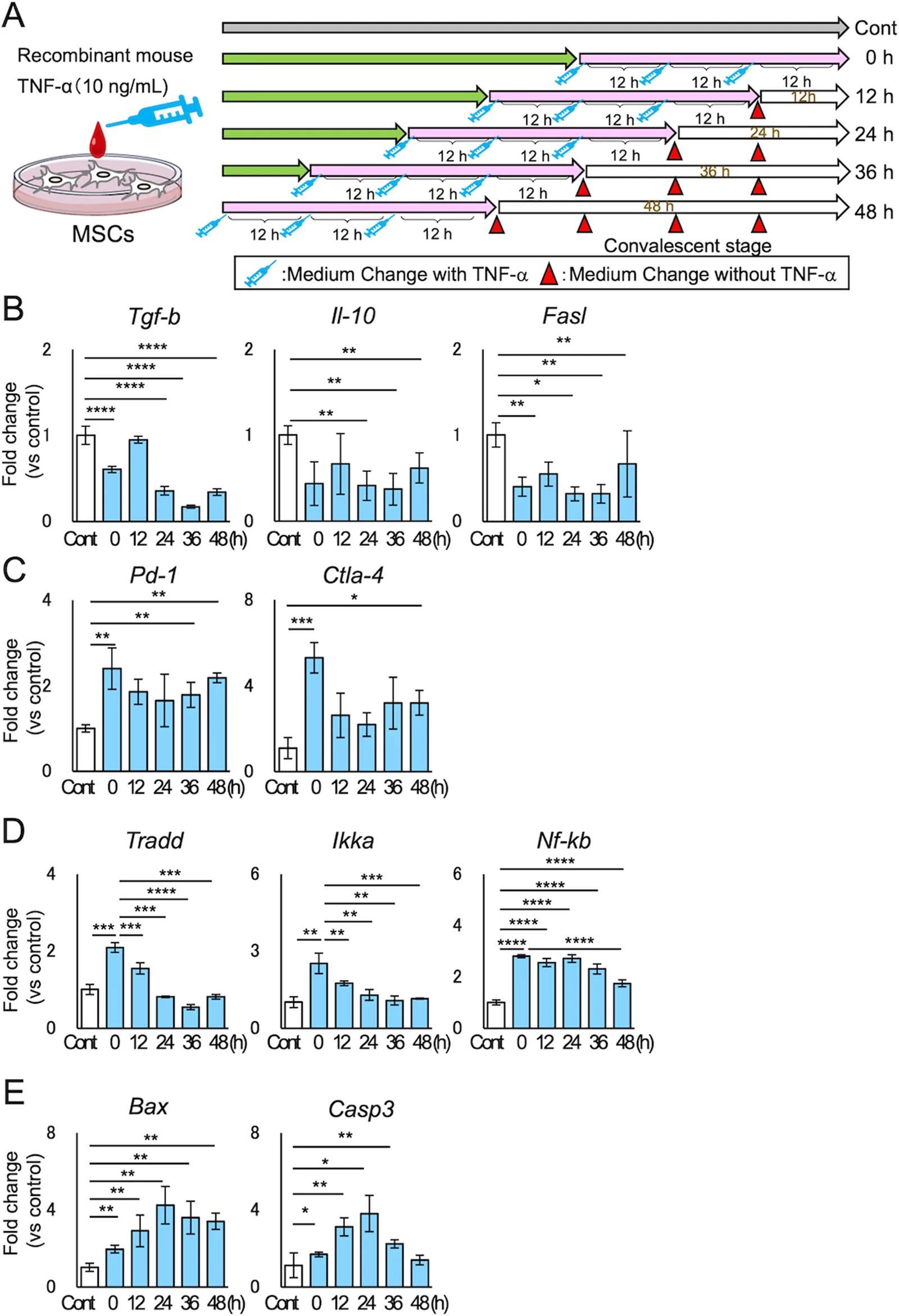
MSC dysfunction persists after withdrawal of TNF-α stimulation. **(A)** Schematic of the withdrawal experiment: MSCs were stimulated with TNF-α for 36 h, followed by culture in TNF-α–free medium. **(B)** Expression of Tgf-β, Il-10, and Fasl remained suppressed after TNF-α withdrawal. **(C)** *Pd-1* and *Ctla-4* expression declined slightly but did not return to baseline. **(D)** Expression of TNF-α signaling genes (Tradd, Ikkα, and Nf-κb) decreased gradually but remained elevated. **(E)** Apoptosis-related genes (*Casp3* and *Bax*) remained upregulated, indicating sustained cellular stress associated with persistent MSC dysfunction. Data represent mean ± SD of independent biological replicates derived from separately prepared MSC cultures (n = 4−5). One-way ANOVA with Tukey’s post-hoc test was used. *p < 0.05, **p < 0.01, ***p < 0.001, ****p < 0.0001.

To evaluate the reversibility of the MSC exhaustion-like phenotype, we cultured cells in TNF-α–free medium after 36 h of TNF-α stimulation and monitored gene expression over time (Figure 4A). Immunoregulatory genes (*Tgf-β*, *Il-10*, *Fasl*) remained suppressed relative to the non-stimulated controls and did not recover to baseline levels (Figure 4B). Although the expression of *Pd-1* and *Ctla-4* showed a gradual decline following TNF-α withdrawal, they remained elevated compared to the baseline. Similarly, the expression of *Tradd*, *Ikkα*, and *Nf-κb* decreased but did not return to control levels (Figure 4C). Furthermore, the expression of apoptosis-associated stress signaling genes *Bax* and *Casp3* was upregulated after 36 h of TNF-α stimulation and remained elevated for at least 24 h after withdrawal (Figure 4D), suggesting sustained apoptotic signaling and persistent dysfunction in the cells. Here, “persistent dysfunction” specifically refers to the sustained suppression of immunoregulatory gene expression (Tgf-β, Il-10, Fasl), together with prolonged activation of inflammatory and stress-associated pathways (Nf-κb, Bax, Casp3), even after withdrawal of TNF-α. To determine whether prolonged TNF-α stimulation affected MSC survival, cell viability was assessed using a CCK-8 assay at 0, 12, 24, and 48 h. No significant changes in viability were observed during this period (Supplementary Figure S8), indicating that the observed exhaustion-like phenotype was not attributable to loss of cell viability under these experimental conditions.

## 4 Discussion

Our findings demonstrated that prolonged exposure to TNF-α induces a persistent reduction in immunoregulatory gene expression in MSCs and promotes the upregulation of immune checkpoint molecules, including PD-1 and CTLA-4. Notably, PD-1, a well-established checkpoint in T cells, is inducibly expressed in MSCs under prolonged TNF-α stimulation (*in vitro*) and sustained inflammatory conditions (*in vivo*) and is associated with diminished immunomodulatory functions. This phenotype closely resembles T cell exhaustion, although a direct causal relationship has not been definitively established. While our findings support the presence of an “exhaustion-like phenotype” in MSCs, we cannot exclude the possibility that aspects of inflammatory adaptation and cellular senescence contribute to this phenotype. Further studies are required to clarify the relative contributions of these mechanisms and to distinguish exhaustion-like functional impairment from senescence-associated changes. Persistent NF-κB activation and sustained expression of apoptotic markers, such as Bax and Casp3, suggest that prolonged inflammatory stimulation imposes lasting stress on MSCs, potentially through epigenetic silencing of immunoregulatory gene loci. Notably, MSC viability remained unchanged during the 48-h stimulation period, suggesting that the observed phenotype reflects functional impairment rather than overt proliferation arrest or cell loss. Importantly, immunoregulatory gene expression failed to recover even after the withdrawal of TNF-α, underscoring the durable impact of prolonged inflammatory signals on MSC identity and function.

Although transient inflammatory stimulation augmented the immunomodulatory capacity of MSCs, consistent with their physiological role in acute wound repair, prolonged TNF-α exposure induced a phenotypic shift toward dysfunction. This trajectory parallels the well-characterized exhaustion-like phenotype observed in chronically stimulated T cells (Philipp et al., 2022). Strikingly, MSCs failed to regain their immunoregulatory function even after cytokine withdrawal or neutralization, implying that apoptotic and/or epigenetic mechanisms may contribute to the sustained maintenance of an exhaustion-like state with incomplete recovery under the conditions examined. We acknowledge that this 48 h stimulation does not fully recapitulate chronic inflammation, but it was chosen because of the viability limitations observed in longer co-cultures. This hypothesis is supported by the sustained elevation of *Bax* and *Casp3*, along with persistent NF-κB signaling. Collectively, these data indicate that the exhaustion-like phenotype in MSCs is not merely a transient adaptive response but rather a deeper and potentially durable shift in functional programming with limited recovery within the experimental timeframe.

Our data (Supplementary Figure S2) support the notion that transcriptional cooperation among NF-κB, STAT3 (Fan et al., 2013), and AP-1 (Fujioka et al., 2004) contributes to the upregulation of PD-1 (Austin et al., 2014) and CTLA-4 (Lei et al., 2024). Previous studies have shown that these transcription factors synergistically regulate PD-1/PD-L1 axis genes, wherein NF-κB directly binds to the PD-L1 promoter and STAT3 and AP-1 act as co-activators in inflammatory contexts (Zou et al., 2020; Zerdes et al., 2018). In parallel, this transcriptional network may also repress the genes responsible for cytokine production and tissue repair, thereby exacerbating the effects of prolonged inflammatory stimulation on MSC function.

In this context, the progressive upregulation of PD-1 observed in our study, along with the downregulation of the *mTORC1* (Raptor) and *mTORC2* (Rictor) genes (Supplementary Figure S3), suggests a mechanistic link between checkpoint activation and metabolic suppression. Additionally, we observed that AMPK phosphorylation was initially enhanced at the measured time points but declined over time (Supplementary Figure S4), indicating an initial metabolic adaptation that eventually fails under persistent stress. This suggests that under prolonged inflammatory conditions, accumulating oxidative stress may progressively impair the metabolic machinery, thereby contributing to the breakdown of energy regulation. Inadequate AMPK activity under prolonged TNF-α stimulation (48 h) may disrupt cellular energy homeostasis and promote a functional exhaustion-like phenotype in MSCs.

The downregulation of Pd-l1 expression (Supplementary Figure S5), together with the functional effects of PD-1 blockade, suggests that PD-1 signaling may influence MSC function through mechanisms that are not strictly dependent on canonical PD-1/PD-L1 cell–cell interactions. Direct experimental evidence for PD-L1–independent or cell-autonomous PD-1 signaling in MSCs was not obtained in this study; nevertheless, this interpretation is conceptually consistent with the findings in T cells, where PD-1 suppresses AKT/mTOR signaling and metabolic fitness (Wang et al., 2024; Wu et al., 2023). To further assess the functional relevance of PD-1 signaling under prolonged inflammatory stress, PD-1 blockade partially restored anti-inflammatory cytokine expression and mTOR pathway–related genes without suppressing sustained NF-κB activation (Supplementary Figures S6A–C). The concomitant reduction in ROS-positive MSCs suggests an improvement in intracellular metabolic and oxidative stress regulation (Supplementary Figure S6D). Together, these findings suggest that PD-1 signaling accompanies MSC dysfunction under prolonged inflammatory stress, while also indicating that PD-1 blockade induces partial rewiring of immunoregulatory and metabolic signaling rather than complete functional rescue. Extending these observations to an MSC–macrophage interaction context, PD-1 blockade during MSC pretreatment partially restored the ability of MSCs to suppress inflammatory gene expression in M1 macrophages in an indirect co-culture system (Supplementary Figures S6E,F). Accordingly, these observations highlight the need for future studies to explore whether modulation of immune checkpoint signaling, metabolic pathways, or epigenetic regulation can influence MSC function under inflammatory conditions.

In our *in vivo* model of chronic inflammation induced by LPS-PG injection, we observed elevated bone marrow expression of *Tnf-α* and *iNos*, a reduction in PDGFRα^+^TGF-β^+^ cells, and an increase in PDGFRα^+^PD-1^+^ cells. Although we did not directly assess the immunoregulatory function of MSCs *in vivo*, these findings are consistent with the emergence of an exhaustion-like phenotype *in situ*. Further *in vivo* functional studies, such as adoptive transfer models, are necessary to validate this interpretation. We also evaluated the effects of TNF-α stimulation on human MSCs (Supplementary Figure S7). Although the upregulation of immune checkpoint genes and downregulation of immunoregulatory genes were not statistically significant in this model, the overall trend was consistent with that observed in the murine MSCs. This discrepancy may be due to the shorter duration of stimulation *in vitro*, which may not fully replicate the chronic inflammatory conditions encountered *in vivo*. Although 10 ng/mL TNF-α induced reproducible MSC responses *in vitro*, this concentration has not been validated to represent pathophysiological levels *in vivo*; therefore, it should be interpreted with caution.

A general limitation of antibody-based intervention experiments is that isotype controls were not included; thus, potential nonspecific antibody effects cannot be fully excluded.

Clinically, our findings highlight the need for caution when considering MSC-based therapies in patients with chronic inflammatory diseases. The inflammatory microenvironment may precondition MSCs for dysfunction, limiting their therapeutic efficacy. Future interventions may require preconditioning regimens, immune checkpoint inhibition, or inflammation-targeted adjuvant therapies to maintain MSC potency after transplantation. Overall, this study identifies the prolonged inflammatory stimulation–induced exhaustion-like phenotype as an underappreciated limitation of MSC-based therapeutics and provides a rationale for developing reprogramming strategies to enhance MSC resilience in hostile inflammatory environments.

## 5 Conclusion

Prolonged TNF-α exposure induces an exhaustion-like phenotype in MSCs, characterized by sustained expression of immune checkpoint molecules and diminished immunoregulatory gene expression. These findings highlight the importance of the inflammatory microenvironment in shaping MSC immunoregulatory states and indicate that immune checkpoint upregulation accompanies inflammation-induced MSC dysfunction. Further studies will be required to determine the mechanistic contribution of PD-1 signaling and NF-κB activation to this altered phenotype.

## Data Availability

The original contributions presented in the study are included in the article/Supplementary Material, further inquiries can be directed to the corresponding author.

## References

[B1] Akiyama K. Chen C. Wang D. Xu X. Qu C. Jin Y. (2012). Mesenchymal-stem-cell-induced immunoregulation involves FAS-ligand-/FAS-mediated T cell apoptosis. Cell Stem Cell 10 (5), 544–555. 10.1016/j.stem.2012.03.007 22542159 PMC3348385

[B2] Austin J. W. Lu P. Majumder P. Ahmed R. Vaziri C. Jin Y. (2014). STAT3, STAT4, NFATc, and CTCF regulate PD-1 through multiple novel regulatory regions in murine T cells. J. Immunol. 192 (10), 4876–4886. 10.4049/jimmunol.1302750 24711622 PMC4011967

[B3] Caires H. R. Barros Da Silva P. Barbosa M. A. Pereira T. Carvalho O. Gomes M. E. (2017). A co-culture system with three different primary human cell populations reveals that biomaterials and MSC modulate macrophage-driven fibroblast recruitment. J. Tissue Eng. Regen. Med. 12 (3), e1433–e1440. 10.1002/term.2560 28865088

[B4] Chen C. Akiyama K. Wang D. Xu X. Qu C. Jin Y. (2015). mTOR inhibition rescues osteopenia in mice with systemic sclerosis. J. Exp. Med. 212 (1), 73–91. 10.1084/jem.20140643 25534817 PMC4291526

[B5] da Silva M. L. Chagastelles P. C. Nardi N. B. (2006). Mesenchymal stem cells reside in virtually all post-natal organs and tissues. J. Cell Sci. 119 (11), 2204–2213. 10.1242/jcs.02932 16684817

[B6] Fan Y. Mao R. Yang J. (2013). NF-κB and STAT3 signaling pathways collaboratively link inflammation to cancer. Protein Cell 4 (3), 176–185. 10.1007/s13238-013-2084-3 23483479 PMC4875500

[B7] Fujioka S. Niu J. Schimidt C. Sclabas G. M. Frederick W. A. Dong Q. (2004). NF-κB and AP-1 connection: mechanism of NF-κB-dependent regulation of AP-1 activity. Mol. Cell. Biol. 24 (17), 7806–7819. 10.1128/MCB.24.17.7806-7819.2004 15314185 PMC507000

[B8] González M. A. Gonzalez R. E. Rico L. Becerra E. Mallo M. Garcia M. A. (2009). Adipose-derived mesenchymal stem cells alleviate experimental colitis by inhibiting inflammatory and autoimmune responses. Gastroenterology 136 (3), 978–989. 10.1053/j.gastro.2008.11.041 19135996

[B9] Gunasinghe S. D. Peres N. G. Goyette J. Thomas B. J. Kershaw M. H. Oliaro J. (2021). Biomechanics of T cell dysfunctions in chronic diseases. Front. Immunol. 12, 600829. 10.3389/fimmu.2021.600829 33717081 PMC7948521

[B10] Horwitz E. M. Le Blanc K. Dominici M. Mueller I. Slaper-Cortenbach I. Marini F. (2005). Clarification of the nomenclature for MSC: the international society for cellular therapy position statement. Cytotherapy 7 (5), 393–395. 10.1080/14653240500319234 16236628

[B11] Lei Z. Tang R. Wu Y. Chen R. Liu Y. Li Q. (2024). TGF-β1 induces PD-1 expression in macrophages through SMAD3/STAT3 cooperative signaling in chronic inflammation. JCI Insight 9 (7), e165544. 10.1172/jci.insight.165544 38441961 PMC11128204

[B12] Mun A. Y. Akiyama K. Wang Z. Lee S. Y. Chen C. Jin Y. (2024). Macrophages modulate mesenchymal stem cell function via tumor necrosis factor alpha in tooth extraction model. JBMR Plus 8 (8), ziae085. 10.1093/jbmrpl/ziae085 39086598 PMC11289833

[B13] Petrini I. Pacini S. Petrini M. Fazzi R. Fazzi C. Fazzi M. (2009). Mesenchymal cells inhibit expansion but not cytotoxicity exerted by gamma-delta T cells. Eur. J. Clin. Invest. 39 (9), 813–818. 10.1111/j.1365-2362.2009.02171.x 19522834

[B14] Philipp N. Kazerani M. Nicholls A. Hou J. Wang L. Kim J. (2022). T cell exhaustion induced by continuous bispecific molecule exposure is ameliorated by treatment-free intervals. Blood 140 (10), 1104–1118. 10.1182/blood.2022015956 35878001 PMC10652962

[B15] Shen Z. Huang W. Liu J. Chen J. Wang Y. Fang Y. (2021). Effects of mesenchymal stem cell-derived exosomes on autoimmune diseases. Front. Immunol. 12, 749192. 10.3389/fimmu.2021.749192 34646275 PMC8503317

[B16] Wang S. Liu C. Yang C. Zhang X. Liu Y. Li M. (2024). PI3K/AKT/mTOR and PD1/CTLA4/CD28 pathways as key targets of cancer immunotherapy. Oncol. Lett. 28, 567. 10.3892/ol.2024.14700 39390982 PMC11465225

[B17] Wherry E. J. Ha S. J. Kaech S. M. Haining W. N. Sarkar S. Subramaniam S. (2007). Molecular signature of CD8+ T cell exhaustion during chronic viral infection. Immunity 27 (4), 670–684. 10.1016/j.immuni.2007.09.006 17950003

[B18] Wu H. Zhao X. Hochrein S. M. Yan Y. Jin W. Xiao Z. (2023). Mitochondrial dysfunction promotes the transition of precursor to terminally exhausted T cells through HIF1α-mediated glycolytic reprogramming. Nat. Commun. 14, 6858. 10.1038/s41467-023-42634-3 37891230 PMC10611730

[B19] Zerdes I. Matikas A. Bergh J. Valachis A. Foukakis T. Kallergi G. (2018). Genetic, transcriptional and posttranslational regulation of the programmed death protein ligand 1 in cancer: biology and clinical correlations. Oncogene 37 (34), 4639–4661. 10.1038/s41388-018-0303-3 29765155 PMC6107481

[B20] Zheng K. Zhen X. Yang W. (2022). The role of metabolic dysfunction in T-cell exhaustion during chronic viral infection. Front. Immunol. 13, 843242. 10.3389/fimmu.2022.843242 35432304 PMC9008220

[B21] Zou S. Tong Q. Liu B. Wu Z. Zhao Y. Wang Y. (2020). Targeting STAT3 in cancer immunotherapy. Mol. Cancer 19 (1), 145. 10.1186/s12943-020-01258-7 32972405 PMC7513516

