# Supplementary material for: Prolonged TNF-α stimulation induces a PD-1–associated exhaustion-like phenotype in mesenchymal stromal cells
Source: Front Cell Dev Biol. 2026 Mar 6;14:1680076. doi: 10.3389/fcell.2026.1680076 (PMC13002830; doi:10.3389/fcell.2026.1680076)
Supplement: Supplementary file 1 [file Supplementaryfile1.docx]

Supplementary Material and Methods

**Prolonged TNF-α Stimulation Induces a PD-1–Associated Exhaustion-Like Phenotype in Mesenchymal Stromal Cells**

**Naoya Matsunaga¹, Kentaro Akiyama²*, Aung Ye Mun¹, Tinling Zou¹, Kazuki Ito¹, Ruji Tagashira¹, and Takuo Kuboki¹**
¹ Department of Oral Rehabilitation and Regenerative Medicine, Okayama University Graduate School of Medicine, Dentistry and Pharmaceutical Sciences, Okayama, Japan
² Department of Occlusal and Oral Functional Rehabilitation, Okayama University Graduate School of Medicine, Dentistry and Pharmaceutical Sciences**,** Okayama, Japan

1. **Supplementary methods**
   1. **Western blot Analysis**

MSCs were lysed in RIPA buffer containing a protease and phosphatase inhibitor cocktail (Sigma-Aldrich, MO, USA). Protein concentrations were determined using the BCA assay (Thermo Fisher Scientific, CA, USA). Equal amounts of protein (20 µg per lane) were separated by SDS-PAGE on 10% polyacrylamide gels and transferred onto PVDF membranes (Cytiva, MA, USA). Membranes were blocked with 5% non-fat dry milk in PBS-T (0.1% Tween-20 in PBS) for 1 hour at room temperature and incubated overnight at 4°C with the following primary antibodies:

- anti-phospho-p-65 (Ser536) (1:500, Cell Signaling Technology, #3033S, MA, USA)
- anti-p-65 (1:500, Cell Signaling Technology, #8242S)
- anti-pAMPK (1:500, Cell Signaling Technology, #2535)
- anti-AMPK (1:500, Cell Signaling Technology, #2532)
- anti–β-actin (1:4000, Sigma-Aldrich, #A5441)

After washing, the membranes were incubated with horseradish peroxidase-conjugated secondary antibodies (1:4000, Cell Signaling Technology) for 1 h at room temperature. The bands were detected using enhanced chemiluminescence (ECL; MilliporeSigma, MA, USA) and visualized using a ChemiDoc MP Imaging System (Bio-Rad). Densitometric analysis was performed using the ImageJ software (NIH, Bethesda, MD, USA).

- 1. **Human MSCs culture**

Human MSCs were purchased (Lonza Basel, Switzerland) and cultured in α-MEM supplemented with 10% FBS, 2 mM L-glutamine, 100 U/mL penicillin-streptomycin, and 55 µM 2-mercaptoethanol (Life Technologies, CA, USA) at 37°C in 5% CO₂. Passage 9 was used for the experiments, as these cells retained stable growth characteristics and were within the supplier-recommended passage range for functional assays.

- 1. **Reactive Oxygen Species (ROS) staining**

To assess the accumulation of reactive oxygen species (ROS) following TNF-α stimulation, cells were stained using a commercial ROS detection kit containing the fluorescent probe DCFDA (2’,7’-dichlorofluorescin diacetate), according to the manufacturer’s instructions (Dojindo Laboratories, Kumamoto, Japan). ROS-positive cells were visualized using fluorescence microscopy, and the proportion of positive cells was quantified using ImageJ software in three randomly selected non-overlapping fields.

**1-4 PD-1 blockade under prolonged TNF-α stimulation**

MSCs were stimulated with recombinant TNF-α (10 ng/mL) for 48 h to induce an exhaustion-like inflammatory response. During stimulation, PD-1 signaling was blocked by adding an anti-PD-1 monoclonal antibody (InVivoMAb anti-mouse PD-1, Bio X Cell) at final concentrations of 4 or 20 μg/mL. The control cells were cultured without anti-PD-1 antibodies. Isotype-matched control antibodies were not included, which is acknowledged as a limitation of the experimental design. After 48 h, total RNA was extracted and subjected to quantitative real-time PCR. The mRNA expression levels of *Tgf-β*, *Il-10*, *Nf-κb*, *Raptor*, and *Rictor* were quantified and normalized to those of the untreated control MSCs. Data are presented as mean ± SD (n = 6). Statistical analysis was performed using one-way ANOVA, followed by Tukey’s multiple comparison test. *Statistical significance was set at p < 0.05*.

**1-5 Cell viability assay after prolonged TNF-α stimulation**

Mouse or Human MSCs were seeded at 4 × 10⁴ cells per well in 96-well plates and cultured overnight. The cells were then stimulated with TNF-α (10 ng/mL) and cultured for the indicated time periods (0, 12, 24, and 48 h). Cell viability was assessed using the Cell Counting Kit-8 (CCK-8) assay, according to the manufacturer’s instructions. Absorbance was measured at 450 nm using a microplate reader, and relative cell viability was calculated by normalizing to the 0 h baseline condition. Each experiment was performed in four replicates and independently repeated three times. Statistical analysis was performed using one-way ANOVA, followed by Tukey’s multiple comparison test. *Statistical significance was set at p < 0.05*.

# Supplementary Table

**2-1 Mouse primers**

**2-2 Human primers**

1. **Supplementary figures**

###
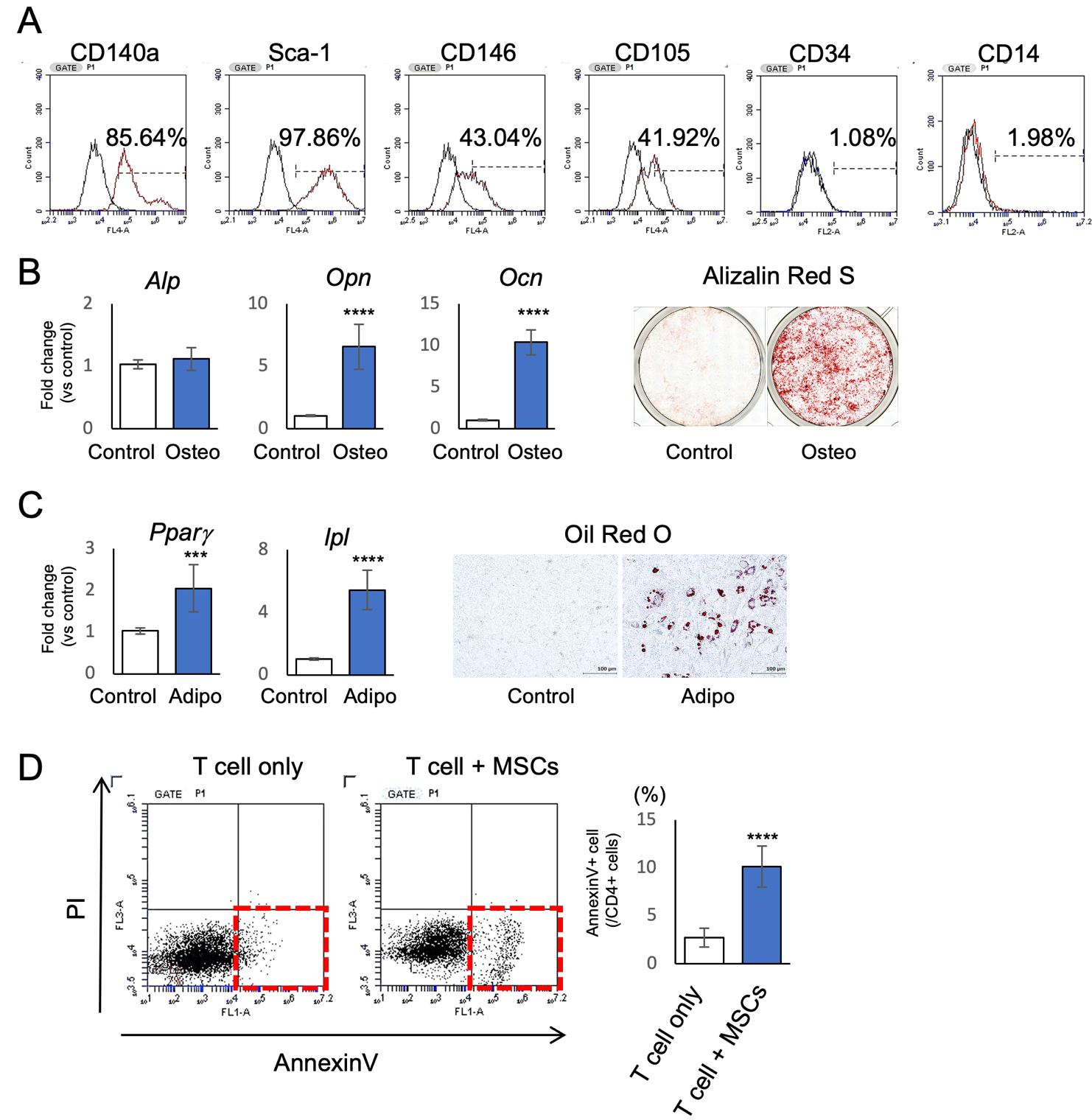


### Supplementary Fig. 1: Characterization and functional validation of mesenchymal stromal

### cells (MSCs).

(A) Flow cytometry analysis of MSC surface marker expression at passage 2. MSCs were positive for CD140a, Sca-1, CD146, and CD105, and negative for hematopoietic markers CD34 and CD14, confirming their typical immunophenotype. (B) Osteogenic differentiation of MSCs. mRNA expression of osteogenic markers Alp, Opn, and Ocn after osteogenic induction compared with that in the control culture. Alizarin Red S staining revealed mineralized nodule formation in the osteogenic group. (C) Adipogenic differentiation of MSCs. The mRNA expression of the adipogenic markers Pparγ and Lpl was upregulated after adipogenic induction compared to that in the control. Oil Red O staining confirmed the accumulation of lipid droplets in the differentiated cells. (D) Immunomodulatory capacity of the MSCs. Co-culture of T cells with MSCs significantly increased the proportion of Annexin V⁺/PI⁺ apoptotic T cells compared to T cells cultured alone, indicating the immunosuppressive effect of MSCs in this functional assay. Data are shown as mean ± SD (n = 5). Statistical analyses were
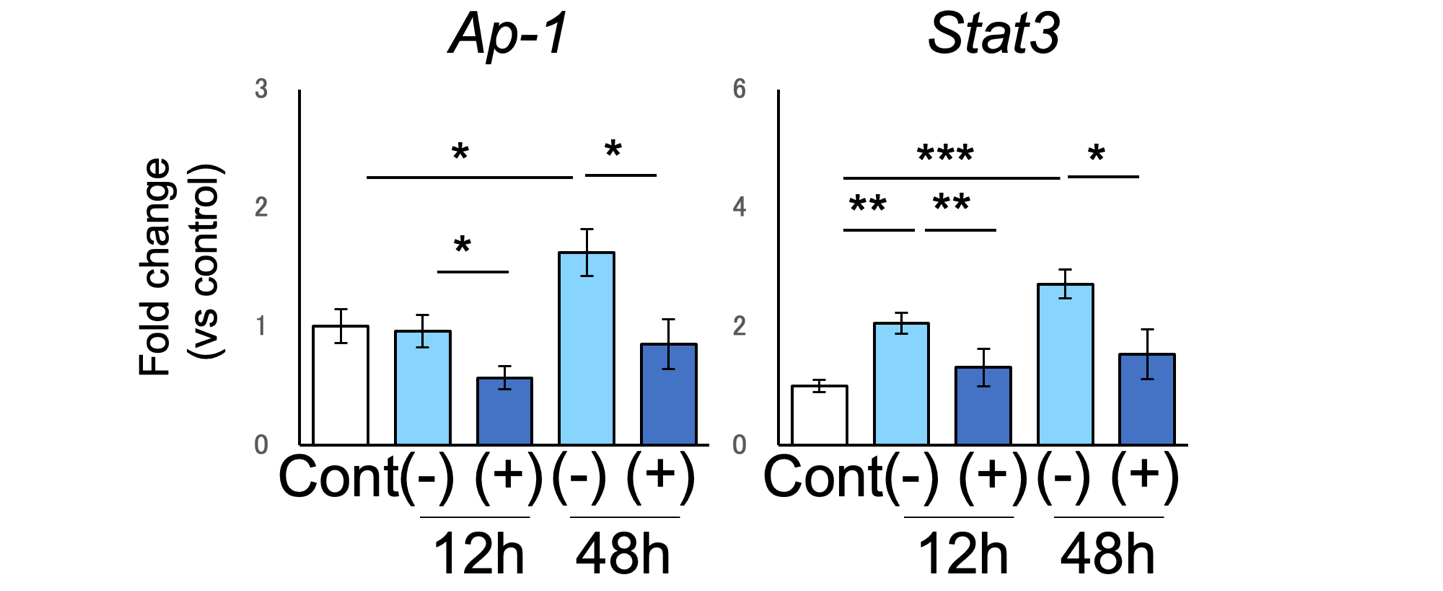
performed using Student’s t-test. ***p < 0.001, ****p < 0.0001.

**Supplementary Fig. 2: Neutralization of TNF-α suppresses M1 macrophage-induced upregulation of *Ap-1* and *Stat3* expression in MSCs.**

mRNA expression of *Ap-1* and *Stat3* in MSCs co-cultured with M1 macrophages for 12 and 48 h, with or without TNF-α-neutralizing antibody. Neutralization suppressed the early upregulation at 12 h and attenuated the subsequent time-dependent changes observed at 48 h. Data are shown as relative expression normalized to the control. Data represent mean ± SD of independent biological replicates derived from separately prepared MSC cultures (n = 4–5). Statistical analyses were performed using one-way analysis of variance (ANOVA), followed by Tukey’s multiple-comparison test. *p < 0.05, **p < 0.01, ***p < 0.001.

**
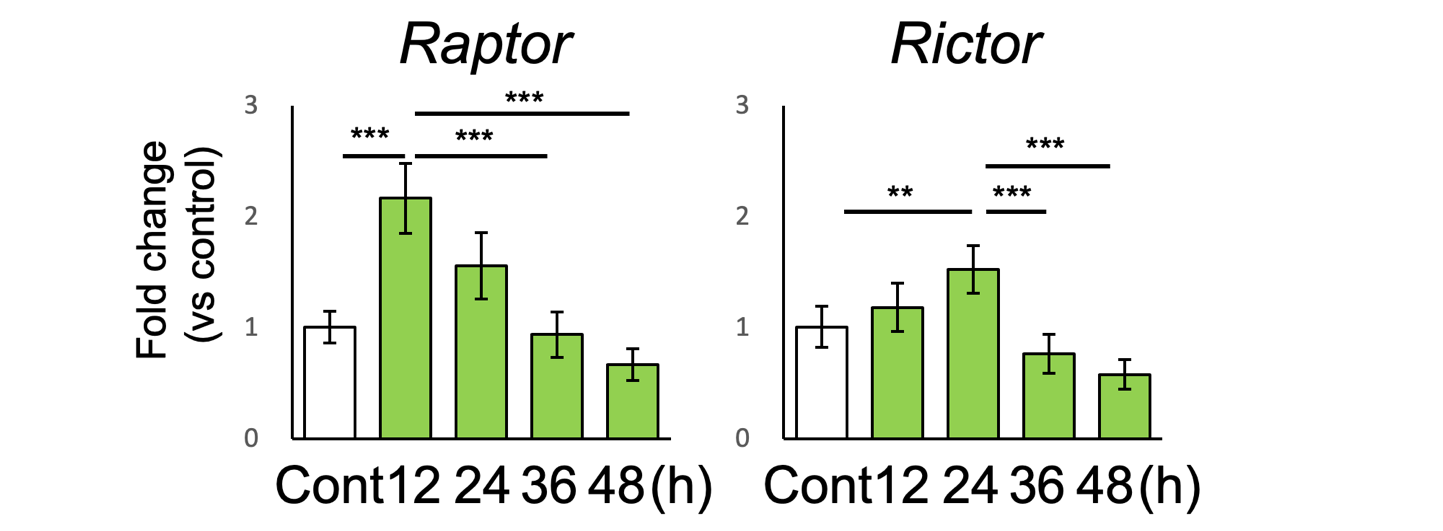
**

**Supplementary Fig. 3: Downregulation of mTOR pathway-related gene expression in MSCs after prolonged TNF-α stimulation.**

The mRNA expression levels of *Raptor* (mTORC1) and *Rictor* (mTORC2) were evaluated using real-time PCR at the indicated time points after treatment. The expression levels were normalized to those of the untreated control MSCs. Data represent mean ± SD of independent biological replicates derived from separately prepared MSC cultures (n = 4–5). Statistical analysis was performed using one-way ANOVA, followed by Tukey’s multiple comparison test. **p < 0.01, ***p < 0.001.


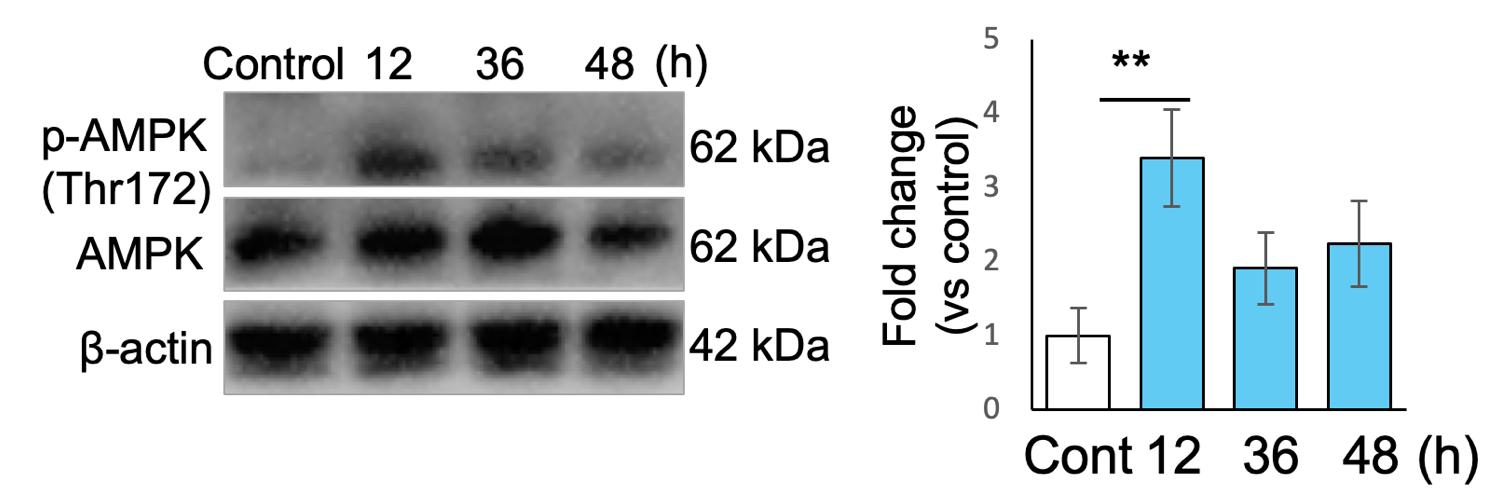


**Supplementary Fig. 4: Transient AMPK activation followed by attenuation during prolonged TNF-α stimulation in MSCs.**

Protein levels of phosphorylated AMPK (p-AMPK) were quantified by western blot analysis at the indicated time points during TNF-α stimulation. The expression levels were normalized to those of the untreated control MSCs. Data represent the mean ± SD of independent biological replicates derived from separately prepared MSC cultures (n = 3). Statistical analysis was performed using one-way ANOVA, followed by Tukey’s multiple comparisons test. **p < 0.01.


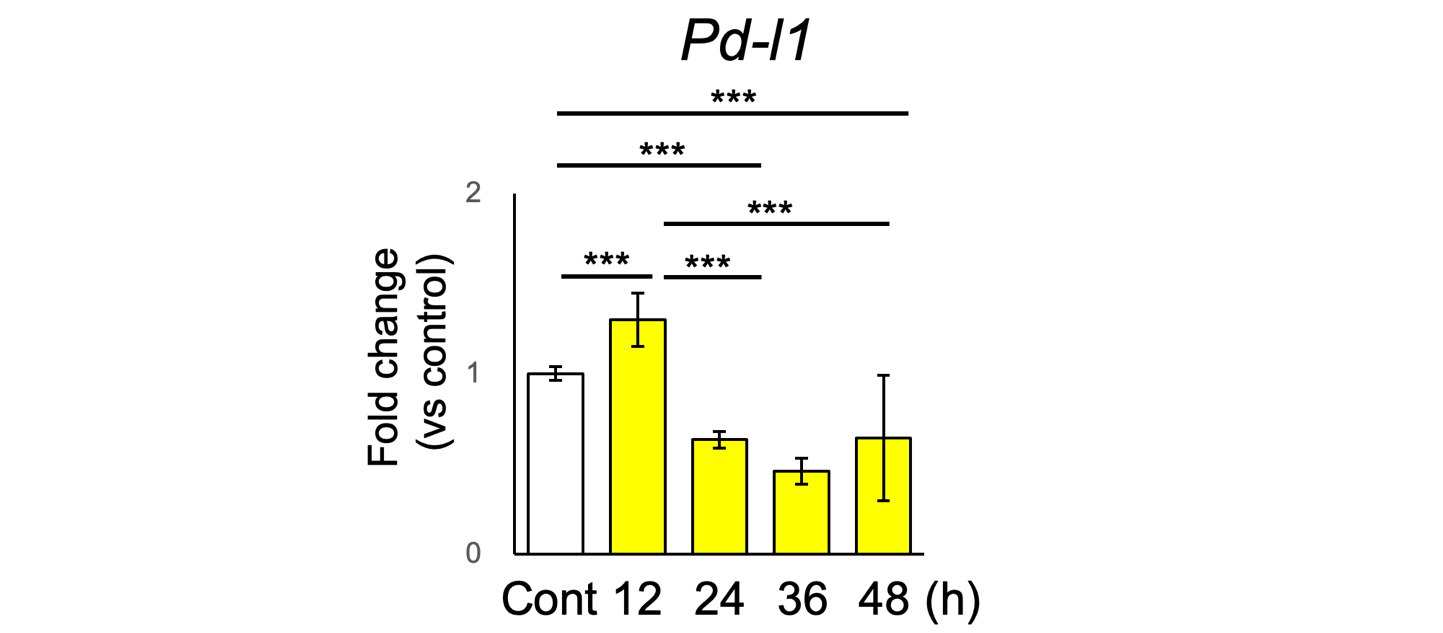


**Supplementary Fig. 5: Downregulation of *Pd-l1* expression in MSCs during prolonged TNF-α stimulation.**

The mRNA expression of *Pd-l1* was quantified by real-time PCR following TNF-α stimulation for 48 h. The expression levels were normalized to those of the untreated control MSCs. Data represent mean ± SD of independent biological replicates derived from separately prepared MSC cultures (n = 4–5). Statistical analysis was performed using one-way ANOVA, followed by Tukey’s multiple comparisons test. ***p < 0.001.


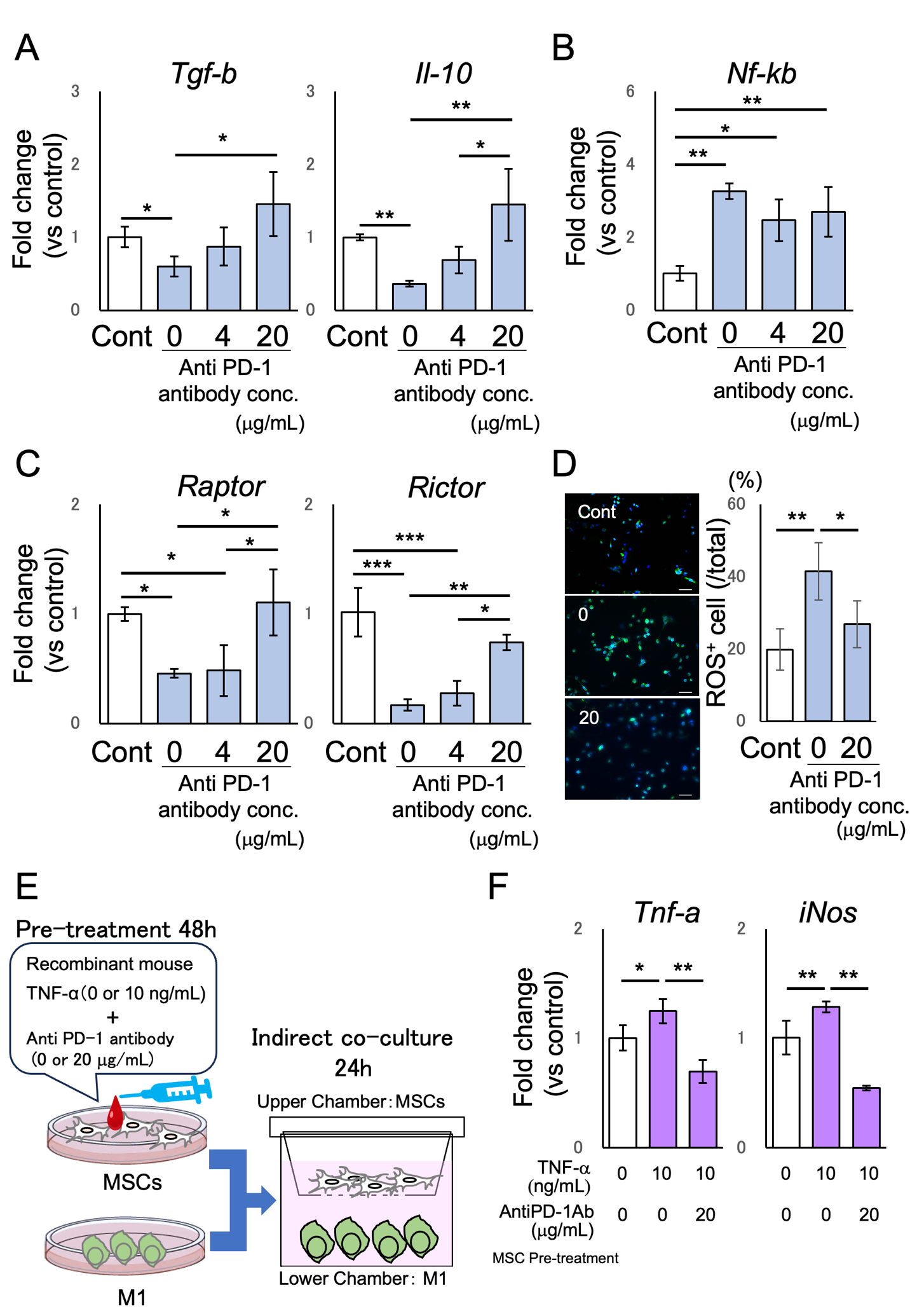


**Supplementary Fig. 6: Functional effects of PD-1 blockade in MSCs exposed to prolonged TNF-α stimulation.**

(A) MSCs were stimulated with TNF-α (10 ng/mL, 48 h) in the presence or absence of anti-PD-1 antibody (0, 4, and 20 μg/mL). mRNA levels of *Tgf-β* and *Il-10* were quantified using real-time PCR and normalized to untreated control MSCs (Cont). (B) *Nf-κb* mRNA expression remained elevated after 48 h of TNF-α stimulation, with no marked decline following PD-1 antibody blockade. (C) mRNA expression of *Raptor* and *Rictor* showed increased expression following PD-1 antibody blockade compared with that in the TNF-α group. (D) Representative images and quantification of ROS-positive MSCs following PD-1 blockade under prolonged TNF-α stimulation. ROS data are presented as supportive evidence consistent with altered intracellular oxidative stress status, rather than definitive mechanistic proof. (E) Schematic representation of the experimental design for indirect co-culture. MSCs were pretreated with TNF-α (10 ng/mL) in the presence or absence of anti–PD-1 antibody (20 μg/mL) for 48 h, followed by indirect co-culture with M1 macrophages for 24 h. (F) mRNA expression of inflammatory genes (*Tnf-α* and *iNos*) in M1 macrophages after indirect co-culture. Data represent the mean ± SD of independent biological replicates derived from separately prepared MSC or macrophage cultures (n = 4-5). Statistical analyses were performed using one-way analysis of variance (ANOVA), followed by Tukey’s multiple-comparison test. *p < 0.05, **p < 0.01, ***p < 0.001.


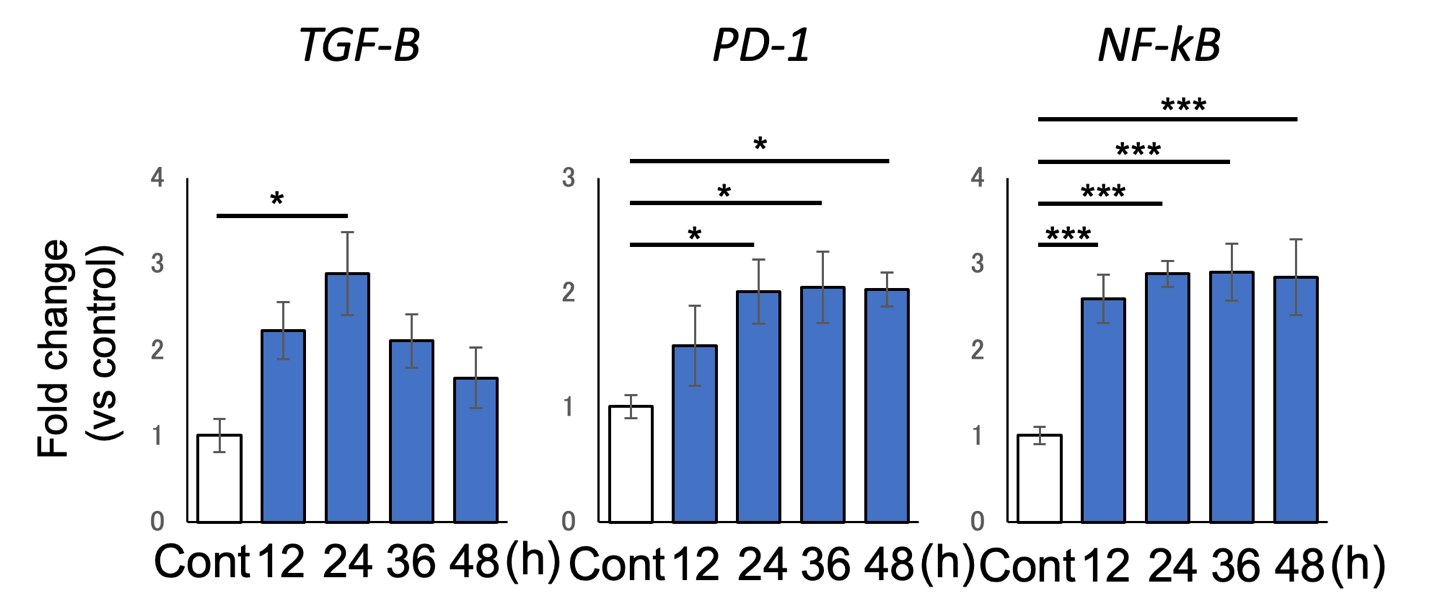


**Supplementary Fig. 7: Upregulation of *PD-1* expression in human MSCs during prolonged TNF-α stimulation.**

The mRNA expression of *TGFB*, *PD-1*, and *NF-kB* was quantified using real-time PCR following TNF-α stimulation for 48 h. The expression levels were normalized to those of untreated control human MSCs. Data represent the mean ± SD of independent biological replicates derived from separately prepared MSC cultures (n = 3). Statistical analysis was performed using one-way ANOVA, followed by Tukey’s multiple comparisons test. *p<0.05, ***p < 0.001.


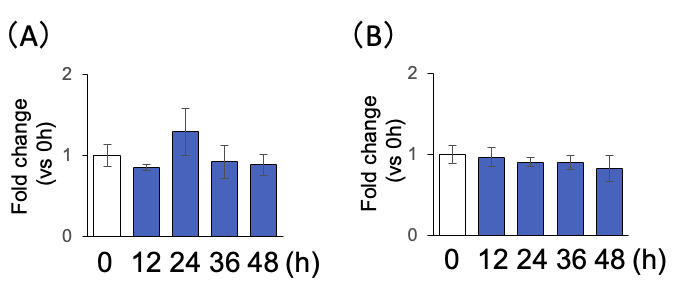
**Supplementary Fig. 8: Cell viability of MSCs during prolonged TNF-α stimulation.**

**
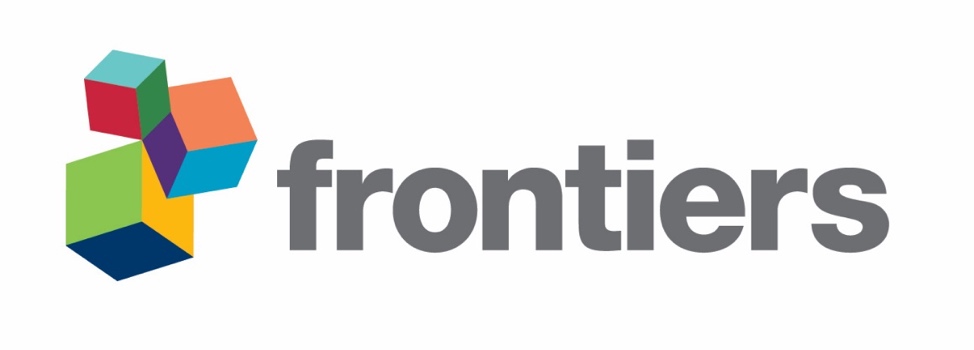
**Mouse MSCs (A) or Human MSCs (B) were stimulated with TNF-α (10 ng/mL) for the indicated time periods (0, 12, 24, and 48 h). Cell viability values at each time point were expressed relative to the 0 h baseline condition. Cell viability was assessed using the CCK-8 assay. Absorbance at 450 nm (OD450) showed no significant change in viability during the stimulation period. Data are shown as mean ± SD (n = 4, three independent experiments). Statistical analysis was performed using one-way ANOVA followed by Tukey’s multiple comparison test.
